# Mine landslide susceptibility assessment using IVM, ANN and SVM models considering the contribution of affecting factors

**DOI:** 10.1371/journal.pone.0215134

**Published:** 2019-04-11

**Authors:** Xiangang Luo, Feikai Lin, Shuang Zhu, Mengliang Yu, Zhuo Zhang, Lingsheng Meng, Jing Peng

**Affiliations:** 1 Faculty Information Engineering, China University of Geosciences, Wuhan, China; 2 Institute of Geological Environment Monitoring, China Geological Survey, Beijing, China; 3 Hubei Jinlang Surey and Design Co., Ltd., Wuhan, Hubei, China; Universidade Federal de Uberlandia, BRAZIL

## Abstract

The fragile ecological environment near mines provide advantageous conditions for the development of landslides. Mine landslide susceptibility mapping is of great importance for mine geo-environment control and restoration planning. In this paper, a total of 493 landslides in Shangli County, China were collected through historical landslide inventory. 16 spectral, geomorphic and hydrological predictive factors, mainly derived from Landsat 8 imagery and Global Digital Elevation Model (ASTER GDEM), were prepared initially for landslide susceptibility assessment. Predictive capability of these factors was evaluated by using the value of variance inflation factor and information gain ratio. Three models, namely artificial neural network (ANN), support vector machine (SVM) and information value model (IVM), were applied to assess the mine landslide sensitivity. The receiver operating characteristic curve (ROC) and rank probability score were used to validate and compare the comprehensive predictive capabilities of three models involving uncertainty. Results showed that ANN model achieved higher prediction capability, proving its advantage of solve nonlinear and complex problems. Comparing the estimated landslide susceptibility map with the ground-truth one, the high-prone area tends to be located in the middle area with multiple fault distributions and the steeply sloped hill.

## 1. Introduction

Mine landslides are common geological hazards that have caused huge loss of life and property worldwide. The loose accumulation of waste slag and lack of stable engineering facilities provide good conditions for development of mine landslides. China is most likely the country with the largest number of heavy mine tailings ponds, and mining activities have produced 20,000 km^2^ of mine tailing wastelands [1]. Hence, the restoration and management of mines are particularly important. Landslide susceptibility modeling (LSM) is considered as a first procedure towards susceptibility assessment, which is a spatial distribution of probabilities of landslide occurrences in a given area based on local geo-environmental factors [2]. Predicting the occurrence of landslide can avoid potential hazards and is helpful for the sustainable development of society [3].

Since the mid-1970s, landslides began to be noticed by many scholars around the world. In recent years, many approaches and techniques have been proposed for landslide susceptibility modeling. Xu et al. [4] used the information value model with seven environmental factors to evaluate debris flow susceptibility. Chen et al. [5] apply information value model using GIS to produce landslide susceptibility map in the Chencang District of Baoji, China. Jie et al. [6] used statistical index and logistic regression model to produce landslide susceptibility maps. Compared with the high subjectivity and difficult reflect nonlinear relationships of statistical models, data-driven models have become popular because of good generalization capabilities. With the development of deep learning, machine learning is back in the spotlight again [7,8]. Peng et al. [9] developed a hybrid model based on the support vector machine (SVM) method to assess landslide susceptibility at the regional scale using multisource data. Binh et al. [10] compared the SVM with other models in landslide susceptibility assessment of Uttarakhand area of India. Bui et al. [11] explored the SVM, artificial neural networks (ANN) and introduced a framework for shallow landslide susceptibility. Pradhan et al. [12] used back-propagation ANN model to assess landslide susceptibility in the Klang Valley area, Malaysia. Conforti et al. [13] built a model based on ANN model to evaluate landslide susceptibility. Moreover, Feng et al. [14] applied the information value model (IVM), logistic regression (LR), ANN and SVM to rainfall-triggered landslide susceptibility mapping.Although there have been many comparison studies on the advantages and disadvantages of these methods in landslide susceptibility mapping, there are few related LSM analyses in the mining field. Su et al. [15] assessed LSM in a coal mine area by using SVM, LR, and ANN models. However, the three fitted models were compared only using the area under the receiver operating characteristics curves (AUC) and some simple evaluation measures, the uncertainty of the models, which is paid growing attention in nowadays, was rarely researched in these studies. On the other hand, the impact of selecting landslide evaluation factors on the study results is also less mentioned. Machine learning algorithms have the ability in dealing with high-dimensional spaces effectively leading to high classification performances. But they do not give a direct way of analyzing the relevance of contributing features [16]. Feature selection methods can be used in combination with machine learning methods to eliminate irrelevant features, give simpler, lower dimensional models while keeping the high classification accuracy.

The main objective of present study is to evaluate and compare the performance of feature selection arithmetic and three assessment methods, including two machine learning models: ANN, SVM and one conventional statistical model: IVM, for mine landslide susceptibility assessment. The uncertainty of the models is analyzed based on the resampling techniques and the rank probability score. For this reason, we extract evaluation factors from remote sensing images and spatial data, which are then represented by three methods, respectively. These models were evaluated using the landslide dataset of Shangli county, China. Analysis of landslide data and model construction have been carried out using ArcGIS 10.2 and Tensorflow 1.2 software. The area with high-prone landslide will be identified and the causes will be discussed in this study.

## 2. Study area

**Fig 1:**
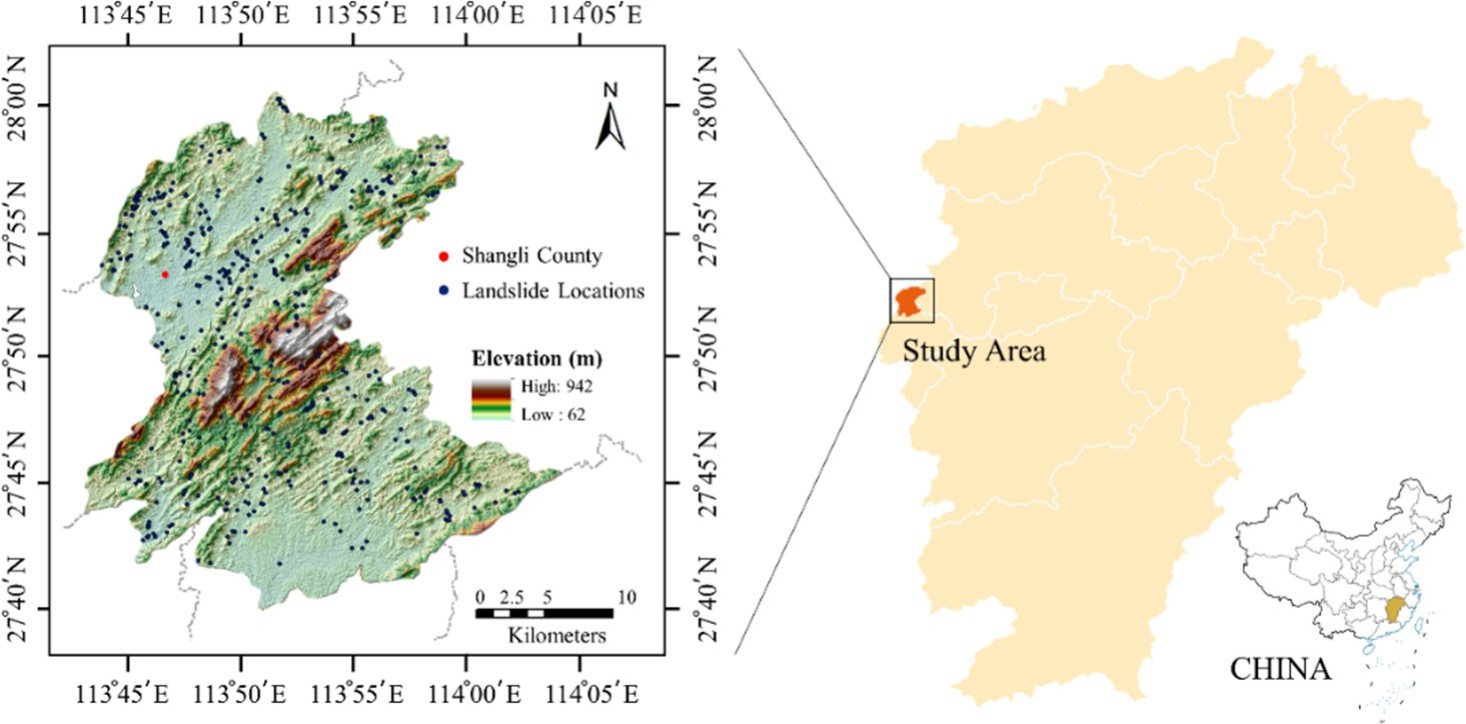
Overview of the study area.

Shangli County was selected as the study area in this paper. This area is located in the middle of China, within longitude 113°43′E−114°04′E and latitude 27°38′N−28°01′N, as shown in Fig 1. It belongs to Jiangxi Province, and the total area is about 720 km^2^. Besides the mountains in the central region, Shangli has rolling hills and valley plain on north and south sides. The area has a distinct four seasons and abundant rainfall. The average annual rainfall is 1300–1700 mm, and the average temperature is 4.8°C in January and 28.7°C in July. The study area is rich in mineral resources and has documented more than 26 minerals. Pingshui river, Lishui river and other tributaries originate from mountains, run across plains and hills, moisten fertile lands, and finally flow into the Xiangjiang River. Topographically, the highest elevation of the study area is 947 m and approximately 45% of the area has a slope angle of less than 20°. Geologically, the main lithology includes sandstone, shale, and limestone rocks.

Landslide inventory is the basis of landslide susceptibility mapping and also affects the accuracy of prediction models. A reliable landslide dataset is crucial for landslide susceptibility modeling. In this study, the landslide inventory was provided by China Geological Survey (http://www.cgs.gov.cn/). Historical landslide inventory, satellite images, and field survey records were used to construct landslides geospatial database. Total 493 locations were prepared for landslide analysis.

There is no clear agreement on the causes of landslides because of their complexity. However, some studies have pointed out several conditioning factors related to landslides, such as topographical, geological, and hydrology conditions. Human activity also has an important effect on the occurrence of landslides. Therefore, based on previous landslide susceptibility studies and analysis of the properties of the study area, sixteen factors were prepared initially for landslide susceptibility assessment: slope angle, slope aspect, elevation, plan curvature, profile curvature, annual rainfall, river density, distance to rivers, lithology, distance to faults, watery degree, road density, distance to roads, the Normalized Difference Vegetation Index (NDVI), the Normalized Difference Water Index (NDWI), and the Urban Land-use Index (ULI).

Slope angle reflects the steepness of mountains and provides a driving force for landslide. Slope aspect and elevation have a great influence on soil, climate and vegetation types, and are related factors of landslide occurrence. Geological structures and faults are also the main predisposing factors of landslides. Theoretically, cracks in rock mass provide favorable conditions for the occurrence of landslides. In addition, roads and rivers can adversely affect stability by eroding the slopes. Therefore, roads and rivers are also chosen as impact factors. NDVI can characterize the vegetation coverage of study area. Rainfall is an important triggering factor of landslides by directly or indirectly reducing the shear strength of rock-soil through physical and chemical effects on rock-soil [10]. Therefore, the mean annual precipitation was selected as the indicator. Dense plant roots can maintain soil to mitigate the effects of rainfall. With the increase of NDVI value, the probability of landslide will decrease gradually, and it is also considered as an important factor of landslide occurrence. The group and classification standard of these data are shown in Table 1.

**Table 1:** Landslide affecting factors and their classes.

Type	Factor	Class
Morphological	Slope angle	(1)<10; (2)10-20; (3)20-30; (4)30-40; (5)>40;
	Slope aspect	(1)N; (2)NE; (3)E; (4)SE; (5)S; (6)SW; (7)W; (8)NW; (9)Flat
	Elevation (m)	(1)<100; (2)100-200; (3)200-300; (4)300-400; (5)400-500; (6)>500;
	Plan curvature	(1)<-0.746; (2)-0.746–0.102; (3)0.102–0.783; (4)>0.783;
	Profile curvature	(1)<-0.910; (2)-0.910–0.007; (3)0.007–0.869; (4)>0.869;
Hydrological	Annual rainfall (mm)	(1)1550-1600; (2)1600-1650; (3)1650-1700; (4)1700-1750; (5)1750-1800;
	River density	(1)0-0.166; (2)0.166–0.477; (3)0.477–0.798; (4)0.798–1.154; (5)>1.154;
	Distance to rivers	(1)0-50m; (2)50-100m; (3)100-150m; (4)150-200m; (5)200-250m; (6)250-300m; (7)>300m;
Geological	Lithology	(1) Cretaceous; (2) Late Yanshanian; (3) Early Yanshanian; (4) Triassic; (5) Permian; (6) Carboniferous; (7) Devonian; (8) Mesoproterozoic Era;
	Distance to faults	(1)<200; (2)200-400; (3)400-600; (4)600-800; (5)>800;
	Soil watery degree	(1)high; (2)middle; (3)low
Other	Road density	(1)<1.015; (2)1.015–1.639; (3)1.639–2.316; (4)2.316–3.513; (5)>3.513;
	Distance to roads	(1)0-50m; (2)50-100m; (3)100-150m; (4)150-200m; (5)200-250m; (6)250-300m; (7)>300m;
	NDVI	(1)<0.2; (2)0.2–0.4; (3)0.4–0.6; (4)0.6–0.8; (5)0.8–1.0;
	NDWI	(1)<0.2; (2)0.2–0.4; (3)0.4–0.6; (4)0.6–0.8; (5)0.8–1.0;
	ULI	(1)<0.2; (2)0.2–0.4; (3)0.4–0.6; (4)0.6–0.8; (5)0.8–1.0;

Maps of landslide affecting factors have been constructed using available data of the study area. Specifically, geomorphological factors namely slope angle (Fig 2A), slope aspect (Fig 2B), elevation curvature (Fig 2C), plan curvature, profile curvature (Fig 2D) have been extracted from ASTER GDEM with 30-meter resolution (https://asterweb.jpl.nasa.gov/gdem.asp) [17]. Annual rainfall data (Fig 2E) has been calculated using 30 years meteorological data (1984–2013) from China Meteorological Data Service Center [18] (https://data.cma.cn/en). Road network (Fig 2F) has been digitalized from traffic map of study area (https://www.google.cn/maps); and then the data of distance to road and road density has been calculated by buffering road sections in the study area [19]. The lithology (Fig 2G) and geological fault line map (Fig 2H) were also vectorized using ArcGIS software. River network (Fig 2I) has been generated from DEM by computing flow accumulation, and then distance to river and river density also has been calculated by buffering river sections [20]. The NDVI (normalized difference vegetation index, Fig 2J), NDWI (normalized difference water index) and ULI (urban land-use index) have been extracted from Landsat 8 imagery using ENVI software (https://landsat.usgs.gov/landsat-8) [21]. The lithology and distance to faults were prepared using a geological map at a scale of 1:200,000 provided by China Geological Survey (http://www.cgs.gov.cn/).

**Fig 2:**
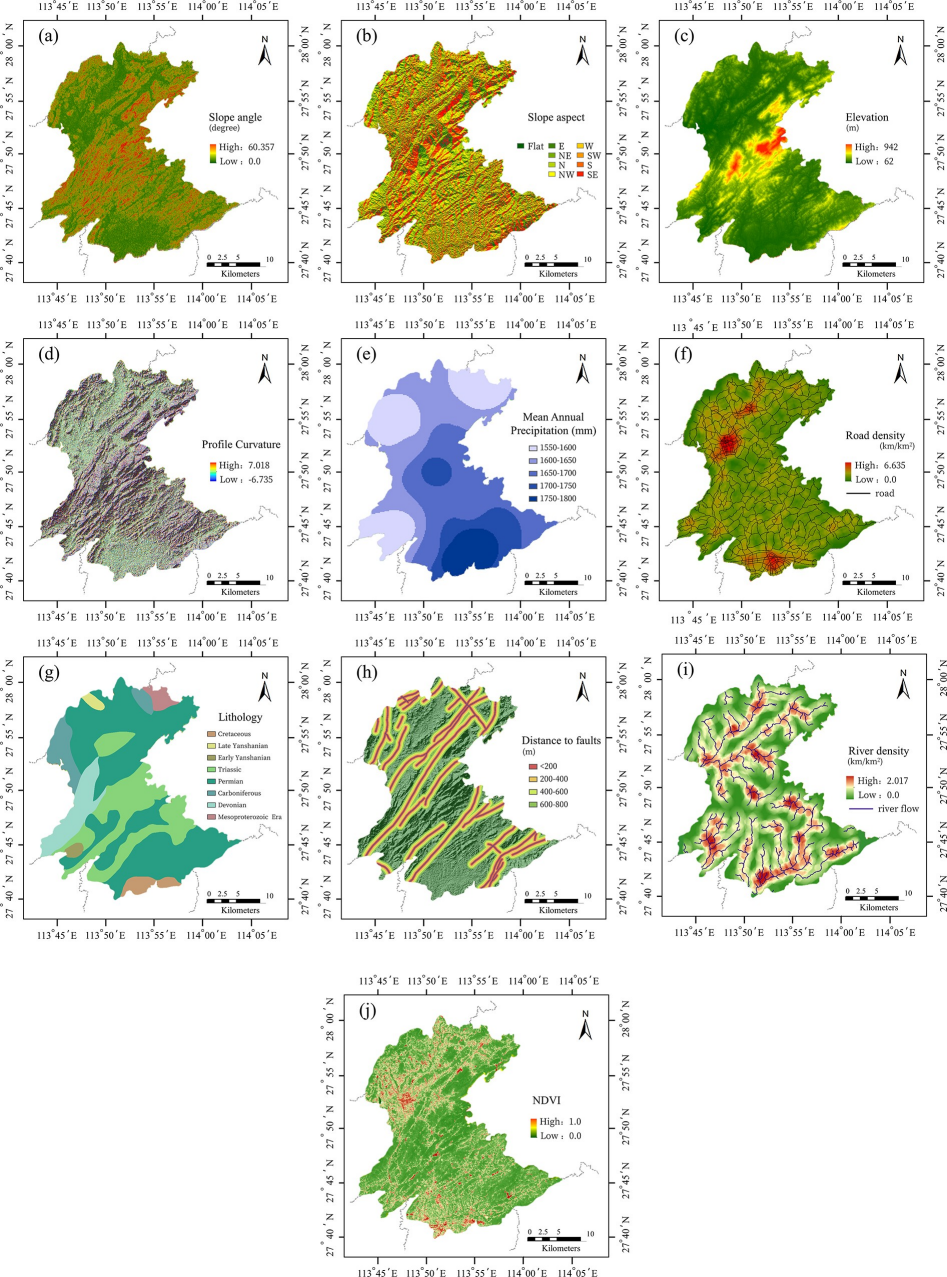
Different affecting factor layer.

## 3. Material and methods

**Fig 3:**
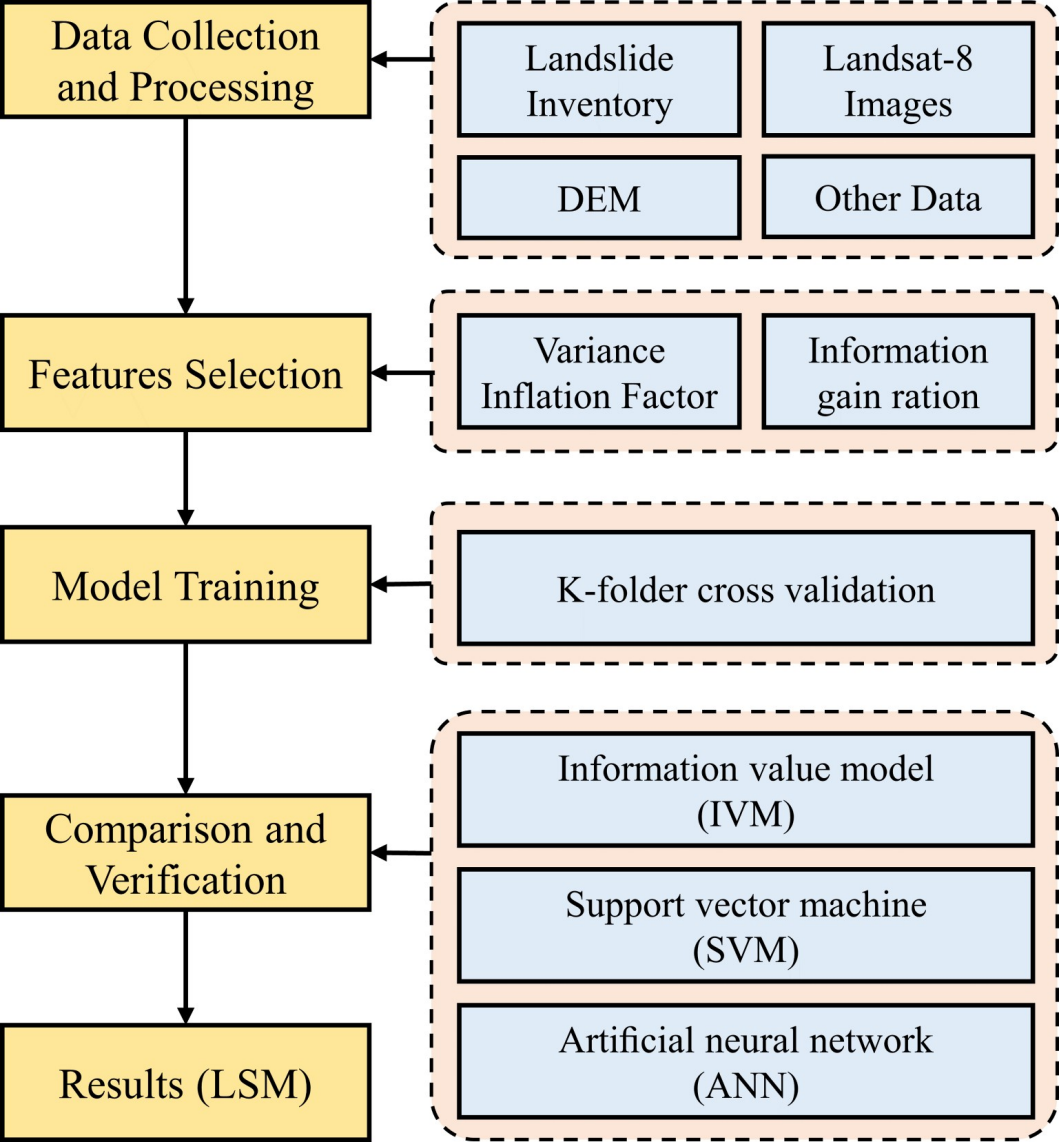
Workflow of the landslide susceptibility analysis.

In this study, landslide susceptibility assessment has been carried out in five steps (Fig 3): (1) collecting and processing data, (2) selecting suitable landslide affecting factors by feature selection method, (3) using K-folder cross validation to divide dataset for model training and testing, (4) constructing and comparing three landslide models, (5) developing landslide susceptibility map of study area. The evaluation measure system contains the receiver operating characteristic curve (ROC), rank probability score and the area percentage of each landslide susceptibility mapping. The objective is to evaluate and compare the comprehensive performance of feature selection arithmetic and three assessment methods for mine landslide susceptibility assessment. The area with high-prone landslide will be identified and the causes will be discussed as a supplement.

### 3.1 Methodology

#### 3.1.1 Artificial neural network

**Fig 4:**
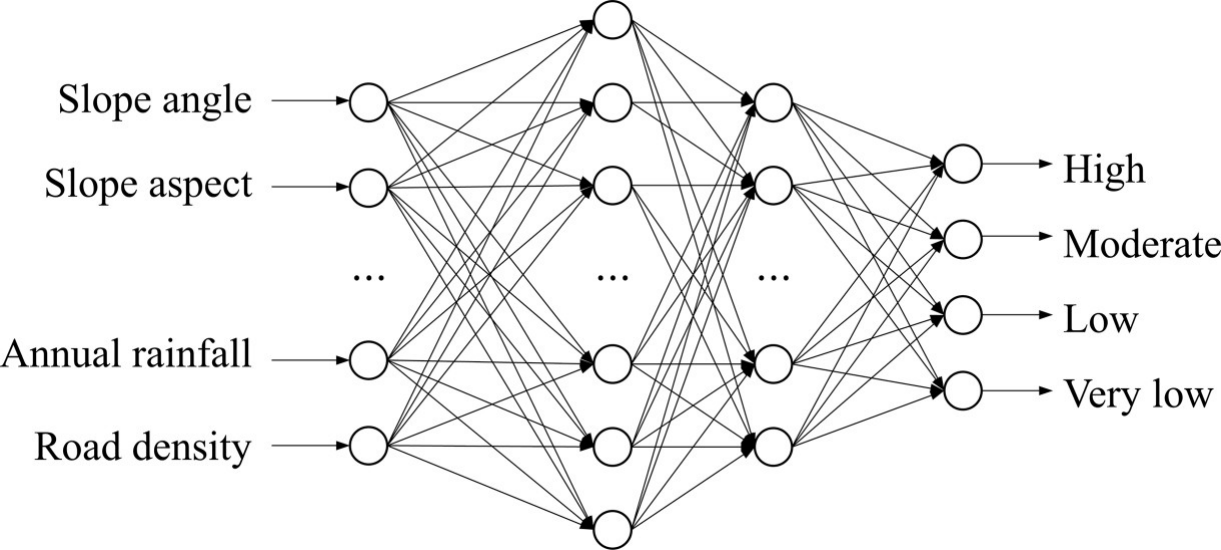
The structure of artificial neural network model.

ANN is a nonlinear computational model that imitate the structure and function of human nervous system [15]. A neural network consists of a large number of artificial neural connections that can be used to estimate or approximate functions. In its classic form, ANN usually contains two layers of input and output layers, and feature transformation is realized by the addition of hidden layers. In this paper, the structure of ANN model is shown in Fig 4.

BP neural network is the most commonly used neural network architecture. The back-propagation algorithm repeats a cycle, including signal propagation and weight update. The signal is propagated forward through the network, layer by layer. Then use the loss function to compare the result with expected output. The error values would be propagated back from the output layer to the input layer, and adjust the weight and threshold of each neuron according to an associated error value.

The learning rate is one of the most important hyper-parameters of ANNs that affect model performance. In present study, the learning rate is calculated by the following formula [22]:

$$\eta (n)=\eta (n-1)*exp(log(\eta_{min}/\eta_{max})/d),$$ (1)

where *η*(*n*) is the learning rate in the nth times training; *η*_*min*_ is the minimum value of the learning rate; *η*_*max*_ is the maximum value of the learning rate, and d is the delay rate.

In this study, the ANN model consists of an input layer, two hidden layer and one output layer. Each neuron in the input layer represents various evaluation factors, while four output layer neurons represent different levels of mine landslide susceptibility, the greater the area affected by the landslide, the higher the landslide sensitivity index is. The neural network is made up by adjusting many parameters including: the learning rate, the momentum factor coefficient, the number of training epochs (iterations) and the Root Mean Square Error (RMSE). The learning rate is a constant controlling the adjustment of the weights associated with the connections, for this analysis was set to 0.01. The momentum factor prevents problems of divergence during research for minimum errors, and was used to accelerate convergence. It was chosen to be 0.5. The number of iterations was set to 8,000, and the RMSE value used for the interrupt of the training phase was set to 0.1.

#### 3.1.2 Support vector machine

SVM is a set of supervised learning methods used for classification, regression and outliers detection [23, 24]. Given labeled training data, the method outputs an optimal hyperplane and classifies new examples [25]. When the input variable is linearly divisible, a method for solving the maximum interval is given, and when the input variable is non-linearly divisible, the original training data set is mapped into a high dimensional feature space by using nonlinear transformation. Then, the SVM model can find an optimal separation hyperplane in the new dimension. Assuming samples (*x*_*i*_,*y*_*i*_):i = 1,2,⋯,n, the optimal hyperplane can be solved by the following function:

$$\{\begin{array}{c} min\left(\frac{1}{2}\|\vec{w}{\|}^{2}+C\sum_{i=1}^{n}\xi_{i}\right)\\ y_{i}\left(\vec{w}\cdot \vec{x}+b\right)\geq 1-\xi_{i}\\ \xi_{i}\geq 0,i=1,2,\cdots ,n \end{array},$$ (2)

where w is the weight vector that determines the orientation of the hyper plane, b is the bias, *ξ*_*i*_ is the positive slack variables for the data points that allow for penalized constraint violation, C is the penalty parameter that controls the trade-off between the complexity of the decision function and the number of training examples misclassified. The function can be converted into an equivalent dual problem based on the Wolf duality theory:

$$\{\begin{array}{l} max\left(\sum_{i}a_{i}-\frac{1}{2}\sum_{i,j}\alpha_{i}\alpha_{j}y_{i}y_{j}\left(\vec{x_{i}}\cdot \vec{x_{j}}\right)\right)\\ s.t.\;\sum_{i}\alpha_{i}y_{i}=0,0\leq \alpha_{i}\leq C \end{array},$$ (3)

where *α*_*i*_ are Lagrange multipliers, C is the penalty. Then, the decision function, which will be used for the classification of new data, can be written:

$$f\left(x\right)=\mathrm{sgn}\left(\sum_{i=1}^{n}y_{i}\alpha_{i}K\left(x_{i},x_{j}\right)+b\right),$$ (4)

where *K*(*x*_*i*_,*x*_*j*_) is the kernel function. The radial basis kernel was adopted as kernel function for SVM model in this study.

SVM method also can be used to solve multi-classification problems, commonly including one-against-one (OAO) and one-against-all (OAA) strategies [26]. The one-against-all approach involves a number of binary classifiers, one for each class. Each binary classifier tries to separate its correspondent class from the other ones, and the multiclassifier output is activated for the class whose binary classifier gives the greatest output amongst all. The one-against-one approach on the other hand constructs a classifier for each pair of classes, resulting in a total of *N*(*N*-1)/2 classifiers. Each classification gives one vote to the winning class and the point is labeled with the class having most votes [27].

#### 3.1.3 Information value model

The IVM is a statistical method based on information theory. In this model, the possibility of landslides occurrence is affected by the information value of factors. The information value I(*x*_*i*_,H) of each landslide predisposing factor *x*_*i*_(*i* = 1,2,…,n) can be expressed as follows [10]:

$$I\left(x_{i},H\right)=\ln\frac{N_{i}/N}{S_{i}/S},$$ (5)

where H represents the likelihood of landslide, *S* is the total number of study units from the study area, N is the total area of landslides in the study area which is the sum of area of all landslide points in the study area, *S*_*i*_ is the number of the study units with the presence of predisposing factor *x*_*i*_, and *N*_*i*_ is the total area of landslides with the presence of predisposing factor *x*_*i*_ which is the sum of area of the landslide points with the presence of predisposing factor *x*_*i*_.

Therefore, the total information I of each study unit can be calculated as the sum of the information values of all predisposing factors.

$$I=\sum_{I=1}^{N}I\left(x_{i},H\right)=\sum_{I=1}^{N}\ln\frac{N_{i}/N}{S_{i}/S},$$ (6)

when I<0, the possibility of landslide occurrence is lower than average; when I = 0, the possibility of landslide is equal to average; and when I>0, the possibility of landslide is higher than average. The larger the information value, the greater the possibility of landslide.

#### 3.1.4 Uncertainty analysis method

In this research, the uncertainty analysis of the models was based on the bootstrap and the rank probability score (RPS). Bootstrap is a popular statistical method that are suitable for small sample. It is a random resampling technique [28] that can expand small samples into large samples, and it will be helpful for calculating the classification probability of each point. Traditional statistical methods like ROC are usually used to assess the classification results right or not, but sometimes, a classification result is neither right nor wrong, it is uncertainty within a range. The rank probability score is a suitable measure for the uncertainty of classification [29]. It can calculate the cumulative error between the predicted category and the actual category. For *K* categories, the RPS defined is as follows:

$$RPS=\sum_{k=1}^{K}{\left(F_{k}-O_{k}\right)}^{2}={\left(F-O\right)}^{2},$$ (7)

where **F** and **O** are cumulative predicted and actual vectors. *F*_*k*_ and *O*_*k*_ are defined as $\sum_{i=1}^{k}F_{i}$ and $\sum_{i=1}^{k}O_{i}$, *F*_*i*_ is the probability that the point is classified into *i* category, if the actual category is *i*, category, *O*_*i*_ = 1, if not, *O*_*i*_ = 0. The closer the RPS is to 0, the better the classification result.

In addition to RPS, RPSS is also used to estimate the performance of models. RPSS is calculated by RPS of predicted classification result and RPS of original data:

$$RPSS=1-\frac{\bar{RPS_{m}}}{\bar{RPS_{o}}},$$ (8)

where $\bar{RPS_{m}}$ and $\bar{RPS_{o}}$ are average RPS values of predicted model and original data. The positive RPSS value indicated that the predicted model is superior to the original model.

### 3.2 Landslide inventory and conditioning factors

Sixteen geo-environmental factors have been initially considered to have an impact on occurrence of landslides in the present study. However, the contribution of each factors to landslide susceptibility models are different. Therefore, it is necessary to evaluate the predictive capability of these landslide affecting factors to eliminate irrelevant or less important factors for further analysis.

#### 3.2.1 Data multicollinearity analysis

Multicollinearity analysis can be used to indicate which factors are redundant with respect to others and improve the accuracy of models. In statistics, the variance inflation factor (VIF) is the reciprocal of tolerance in a model with multiple terms, divided by the variance of a model with one term alone. The VIF and tolerances are both widely used to measure the multicollinearity among factors, a Tolerance of less than 0.2 or a VIF above 5 all indicates a multicollinearity problem [13]. As shown in Table 2 (Before), there is a collinearity between plan curvature and profile curvature. In addition, the VIF value of NDVI, NDWI and ULI are also greater than 5. Therefore, eliminate less important factors needs to be consider further to reduce collinearity between variables.

**Table 2:** The variance inflation factors and tolerances multicollinearity analyze of factors.

Factor	Before		After	
	Tolerance	VIF	Tolerance	VIF
elevation	0.641	1.559	0.642	1.559
slope aspect	0.944	1.060	0.956	1.046
slope angle	0.708	1.412	0.709	1.411
plan curvature	0.085	11.744	-	-
profile curvature	0.086	11.656	0.601	1.664
mean annual rainfall	0.884	1.131	0.900	1.111
distances to faults	0.842	1.188	0.849	1.178
distance to rivers	0.494	2.026	0.489	2.043
distance to roads	0.730	1.370	0.494	2.025
river density	0.488	2.047	0.743	1.345
road density	0.703	1.423	0.707	1.414
NDVI	0.015	67.736	0.809	1.235
ULI	0.010	95.424	-	-
NDWI	0.043	23.519	-	-
lithology	0.723	1.383	0.736	1.359
soil watery degree	0.810	1.235	0.815	1.227

#### 3.2.2 Elimination of the less important factors

As mentioned in Section 3.2.1, we need to eliminate some factors to reduce the multiple collinearity. Obviously, plan curvature and profile curvature are grouped as indicators of terrain fluctuation. NDVI, NDWI and ULI also are grouped as indicators for describing land use. In this paper, we use information gain ratio as the basis of judgment for factor selection. Impurity of information can be measured by information entropy to quantify the uncertainty of predicting the value of the goal variable. The information gain is the change in information entropy H from a prior state to a state that takes some information as given:

IG(T,a)=H(T)−H(T|a), (9)

In this experiment, we use the following formula to calculate.

$$H(T)=-\sum_{k=1}^{K}P\;\log P,$$ (10)

$$H(T|a)=-\sum_{k=1}^{K}\frac{\left|D^{k}\right|}{\left|D\right|}H(T^{k}).$$ (11)

Each evaluate factor was divided into K categories according to Table 1. P in Formula 10 represents the proportion of different mapping level to all landslide records, |*D*| is the all landslides record number, and |*D*^*k*^| is the number of categories K of one of these evaluation factors. However, in Formula 11, H(*T*^*k*^) was use the proportion of different mapping level to this categories number.

Information gain ratio (IGR) is a ratio of information gain to the intrinsic information. IGR is widely used in high dimensional data and is an effective measure to determine the relevance of feature for classification. Not all features contribute equally to landslide occurrence. The feature with a higher value of IGR indicates a higher prediction ability of the models. The importance of features towards decision making in our model is done by evaluating them with the IGR measurement [30]. Hence these features can be sorted in the order of their contribution by listing scores of IGR.

**Fig 5:**
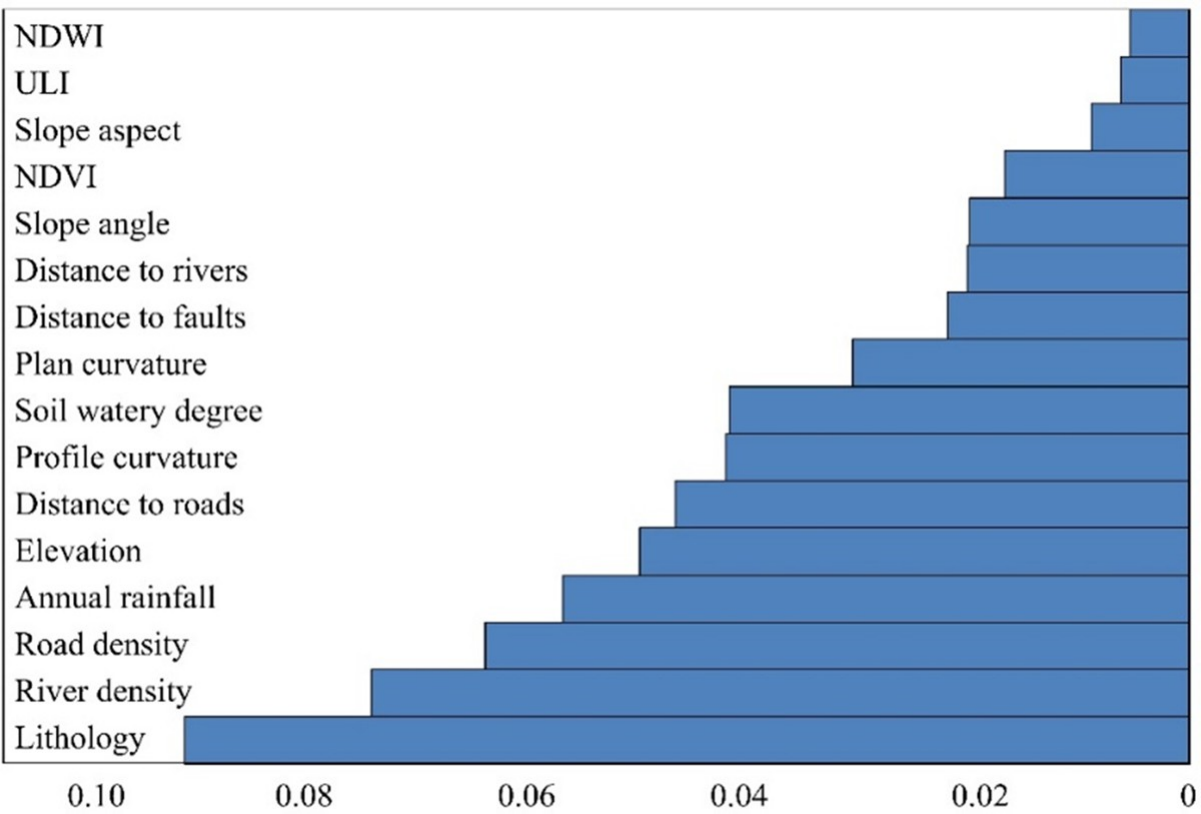
Features sorted in the order of scores of information gain ratio.

The IGR of each factor is shown in Fig 5 and the less important factors (plan curvature, ULI and NDWI) in multicollinearity factor pairs were eliminated to reduce multicollinearity. As shown in Table 2 (After), only 13 factors were used for model construction in the end.

## 4. Discussions

### 4.1 Landslide susceptibility modeling

In the present study, two machine learning models of ANN and SVM and one statistical model of IVM were applied to assessing the landslide susceptibility. After eliminating the less important features, thirteen features, namely slope angle, slope aspect, elevation, profile curvature, mean annual rainfall, river density, distance to rivers, lithology, distances to faults, soil watery degree, road density, distance to roads and NDVI were used as inputs of landslide modeling.

The landslide inventory contains a total of 493 records. Based on coordinates of mines and landslides, the distance between them is calculated and the number of landslide disasters around each mine is counted. According to the number of disasters, the landslide risk grades are divided into four categories, which are high, moderate, low and very low in order. Due to the data imbalance of different categories, in the experiment, oversampling was performed on minority categories, and random noise was added to minority categories to enhance model robustness. In order to make full use of data, the landslide models were constructed using the aforementioned dataset with 5-fold cross validation. For each round, 80% of the data is used for training and 20% for testing. The training dataset is used to train landslide models whereas the testing dataset is utilized to validate the performance of the landslide prediction.

In this study, the ANN and SVM model were constructed by using TensorFlow (https://www.tensorflow.org/). For SVM, after many trial and error processes, we obtained the optimal parameters of the model. Polynomial kernel function was selected as kernel function, and the Penalty coefficient C is 1.0, the gamma is the reciprocal of the number of features, the degree is 3 and the coefficient of kernel function is 0.5.

The information value of factors was calculated according to Eq (5). Then, ArcGIS map algebra tool was used to cover all landslide factors to calculate the total information. Finally, Jenks natural breakpoint method was used to reclassify the total information to generate landslide sensitivity map.

### 4.2 Model validation and comparison

As a useful tool, the receiver operating characteristic curve (ROC) has been widely used to validate the performance of landslide susceptibility models. The ROC usually has a true positive rate on the Y-axis, with a false positive rate on the X-axis at various threshold settings. The false positive value along the x-axis is the proportion of the area divided into landslide prone areas but is actually not (AUC) ranging from 0.5 to 1 [31]. Having a maximum AUC close to 1 indicates that the model produces excellent results. In contrast, an AUC value close to 0.5 means poor results. It is generally considered that if the AUC of the model is greater than 0.7, the model has high accuracy.

**Fig 6:**
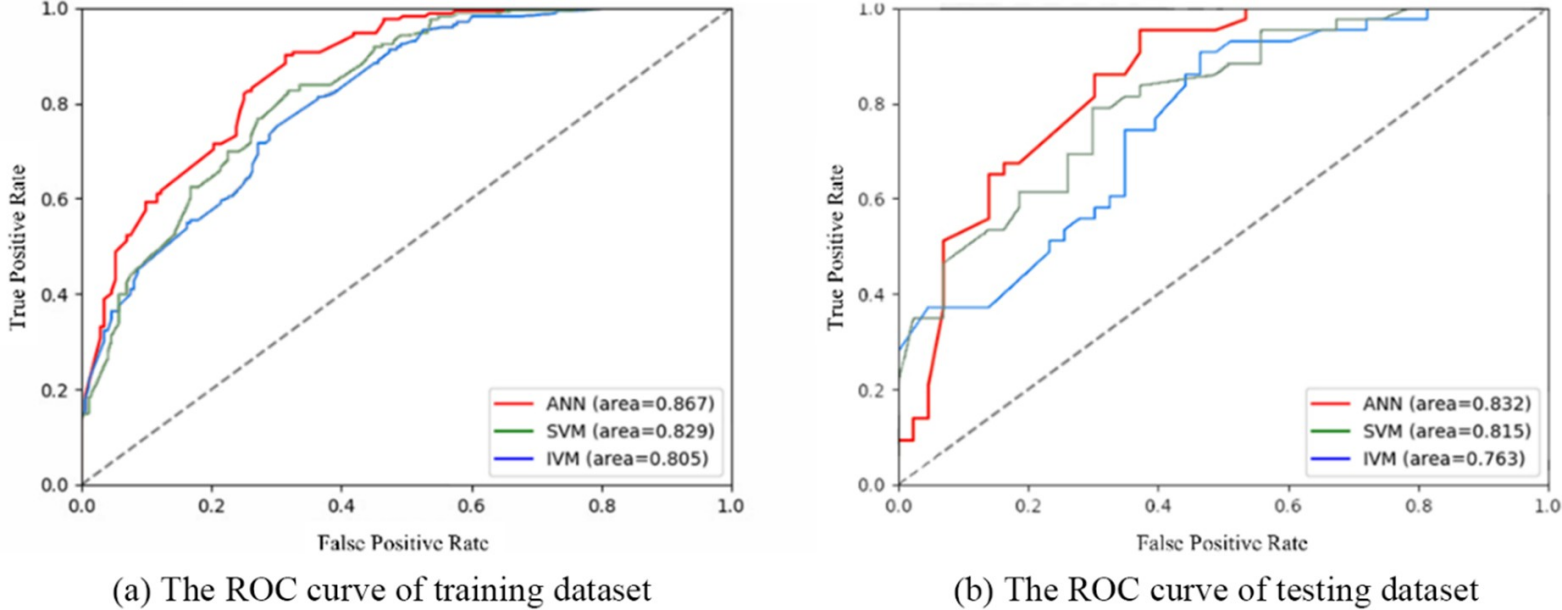
The ROC curves of ANN, SVM and IVM in the training and testing datasets.

The ROC curves in Fig 6 show the training and testing performance of different methods in the landslide modeling. The results show that all applied models have shown good capability for spatial prediction of landslides. The machine learning models of SVM and ANN achieved good performance in both of the training and testing dataset assessment. Out of these, the ANN model has the highest performance, followed by the SVM and IVM model. The mean AUC value of three models is 0.867, 0.829 and 0.805, respectively in training dataset, 0.832, 0.815 and 0.763, respectively in testing dataset. The ANN model achieved higher prediction capability, because it is a powerful data-driven, self-adaptive, and flexible computational tool to solve nonlinear and complex problems.

From Fig 6 We can draw the following points:

1. Based on 13 evaluation factors, all three models showed good performance in landslide susceptibility assessment. The AUC values of ANN, SVM and IVM were 0.867, 0.829 and 0.805 respectively on the training dataset. Even though the AUC value of testing dataset is generally lower than training dataset, it still remains around 0.8;
2. Obviously, ANN model is superior to the other two models in both training data set and verification data set, followed by SVM, and IVM. Among these method, it can be seen that the ANN model has a good fitting effect on the non-linear function relations such as landslide susceptibility evaluation;
3. The ROC curves of three models have similar changes, that is, if one of these model's AUC values increases, the rest models will also increase. The ANN model starts with the lowest TPR and is more evident in the verification data set, but after a period of time it can surpass the other two models and give priority to higher levels, reflecting its rapid adjustment ability.

**Fig 7:**
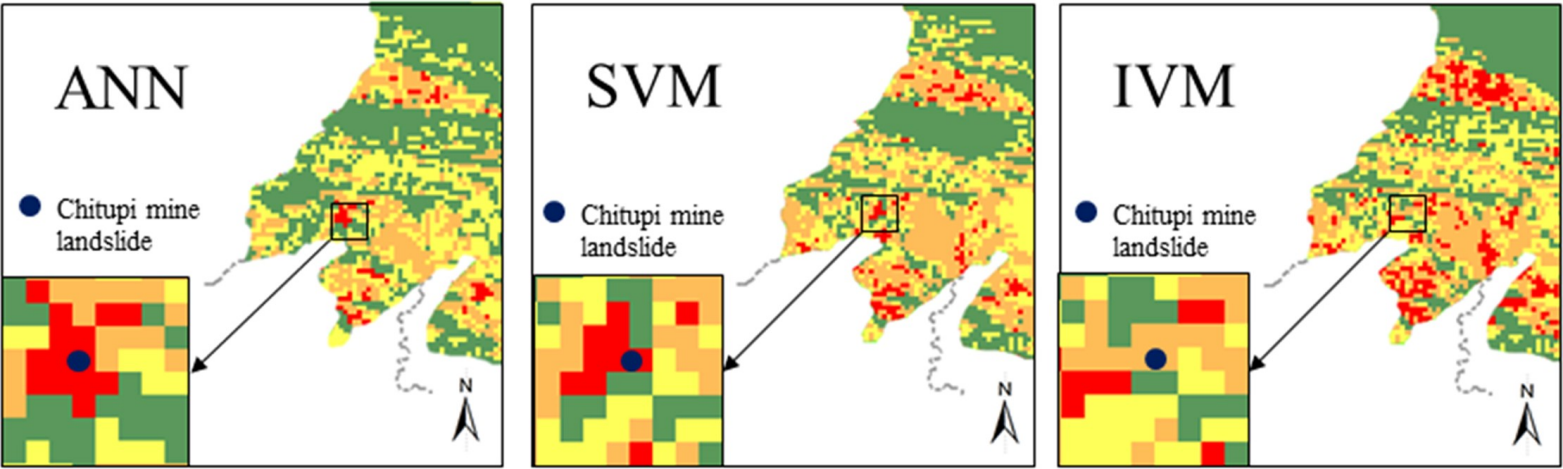
The Chitupi mine landslide estimated result in different models.

**Fig 8:**
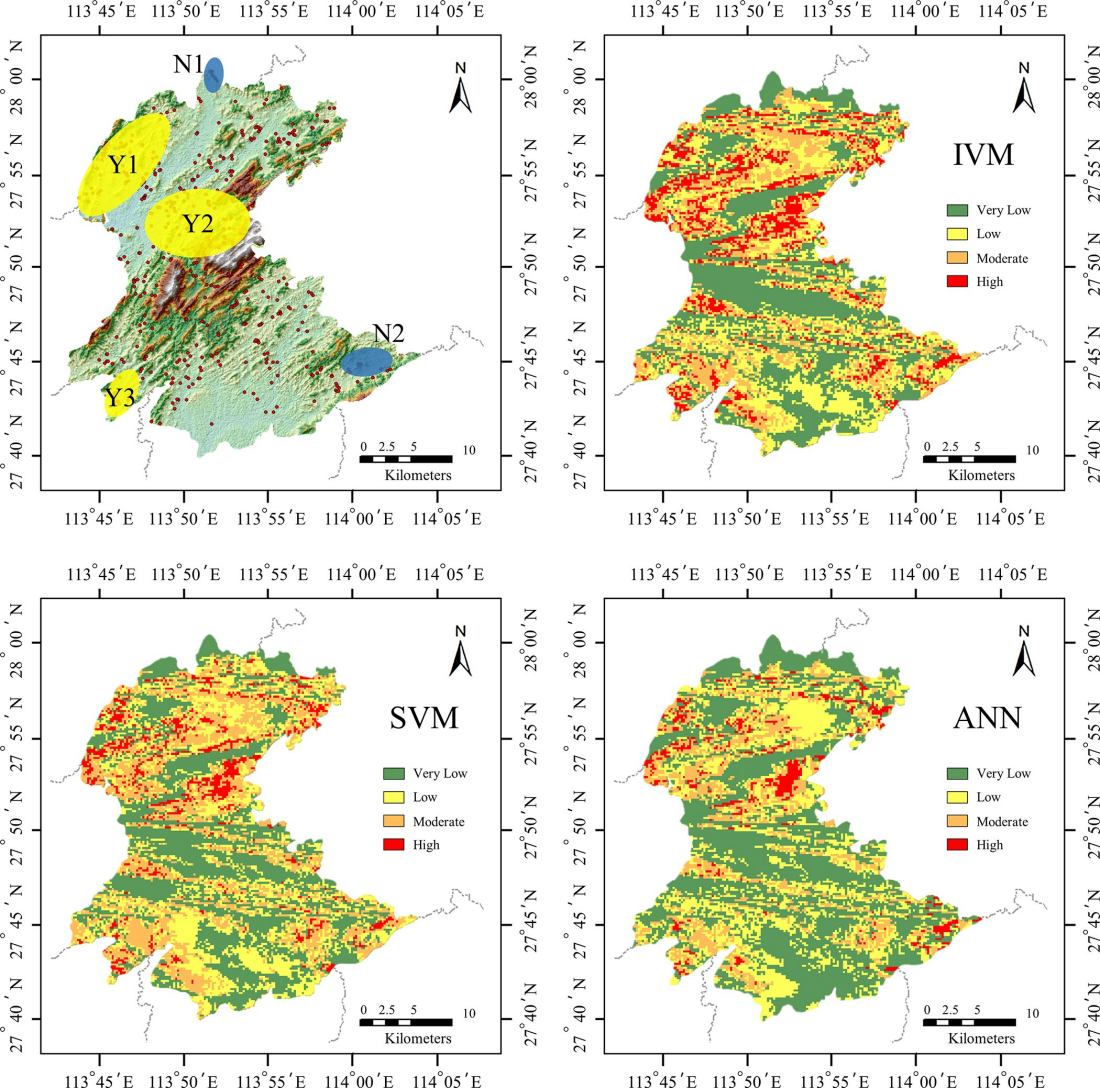
Comparison of actual distribution of landslides with three estimated models.

The Chitupi mine landslide is located in Changping town, Shangli city. The stability of the slope is poor, and the continuous heavy rainfall is very easy to induce the medium landslide. According to the result of field survey, this slope has poor stability and is highly dangerous under continuous heavy rainfall. From Fig 7, we can see that the prediction results also prove the effectiveness of these models. The constructed models were applied to calculating landslide susceptibility indexes for all pixels in the study area. Thereafter, landslide susceptibility maps were prepared by ArcGIS and classified into four classes with ratings of very low, low, moderate, and high susceptibility, which is shown in Fig 8.

As can be seen from Fig 8, the actual distribution of Y1, Y2 and Y3 with more landslide disasters has been well identified in the three estimated models. For the Y1 region, the fault line is densely distributed, and the geological conditions are not stable. In the Y2 region, the terrain is steep, which is not conducive to soil and water conservation, and is prone to landslide under heavy rainfall conditions. For the Y3 region, there are more rivers distributed, and the erosion of the river also makes these areas prone to landslides. On the other hand, there are two regions (N1, N2) in the estimated landslide susceptibility map that do not match ground-truth one. The possible reason for this result is that the rock strata in these areas are relatively hard, and the road network is sparse, which has little damage to geological structure, thus affecting the effect of the model.

**Fig 9:**
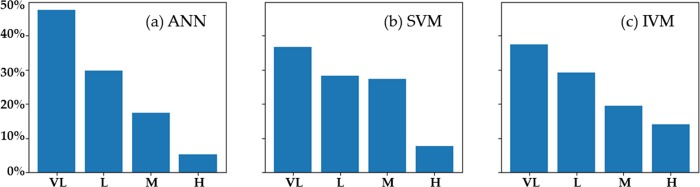
Percentage of each level mapping under different models, VL: Very low, L: Low, M: moderate, H: High.

Fig 9 is obtained by calculating the area percentage of each landslide susceptibility mapping level under different models. Compared with other two models, the ANN model can identify larger very-low risk areas and smaller high-risk areas. This would save a lot of risk prevention costs in specific engineering practices. The IVM model tends to overestimate the risk of a region.

Comparing the estimated landslide susceptibility map with the ground-truth one, it can be found that the high-prone area has multiple fault distributions. These areas are mostly distributed in the middle of the study area. The distribution of minerals is often controlled by fault structure, and the place where the faults suddenly change is a good place for mineralization. Therefore, these areas have a high susceptibility to mine landslides. In the southern part of study area, although the roads are dense, and the vegetation coverage is less, the landslide disasters are not easy to occur because of the gentle terrain and good economic development.

### 4.3 Model uncertainty analysis

The RPS and RPSS of SVM and ANN are shown in Table 3. The IVM model did not perform as well as ANN and SVM referring to AUC values, so it does not participate in the comparison. $\bar{RPS_{m}}$ represents the average RPS values of the ANN and SVM assessment model and $\bar{RPS_{o}}$ is reference model. The closer the RPS is to 0, the better the classification result. A positive RPSS value indicates that the forecast model is superior to the reference forecast. Form the results, it can be found that ANN has a lower RPS value than SVM which indicates that the classification results of ANN have a smaller range of uncertainty and are more reliable. Compare to the values of RPSS, the ANN model and SVM model are both perform better than reference model, and ANN model is superior to the SVM model and original model.

**Table 3:** The RPS and RPSS values of ANN and SVM.

	$\bar{RPS_{m}}$	$\bar{RPS_{o}}$	RPSS
ANN	0.455	0.604	0.25
SVM	0.526	0.604	0.13

## 5. Conclusions

Mine landslide susceptibility assessment is a key step in reducing disaster risk in landslide-prone areas, especially for the restoration of abandoned mines. In this study, Shangli county was taken as a case study where more landslide disasters occurred. This study applied three widely used models including ANN, SVM and IVM to mine landslide susceptibility mapping under totally thirteen affecting factors.

Firstly, models based on machine learning methods show better performance than traditional statistical model. Both the ANN and SVM models have an AUC value of over 80% on training and testing datasets. Overall, the ANN model has best performance, followed by SVM and IVM. This has also been observed by other landslide studies [32]. The IVM model is simple to calculate but does not have good generalization capabilities and tends to overestimate the landslide susceptibility to indicate a higher level. In contrast, the ANN model requires multiple search for optimal parameters.

Obviously, the predictive capability of evaluation factors affects the performance of predictive models. This paper shows that there are only thirteen factors have better predictive capability hence these factors have been used for model construction. Plan curvature and profile curvature, NDWI and ULI can be retain one for each group because of the strong correlation, which can be guessed from the similarity of their formulas. This can provide a reference for work of others.

For the study area, it can be seen from the landslide susceptibility map that the northern part of study area is highly prone to mine landslide disasters due to geological conditions. Although the population activity is dense in the southern area, the susceptibility is still low. As an attempt, although there are uncertainties, it is valuable for engineering practice to get smaller high-risk areas based on optimized assessment model.

In recent years, deep learning technology has developed rapidly. Due to its extremely high classification accuracy, it has been successfully applied in many fields, like human perceptions and environmental simulation [33,34]. In the landslide susceptibility research, deep learning method has been proved to be a reliable method. Xiao et al [35]. compared the performance of deep learning method Long Short Term Memory (LSTM) with the traditional machine learning method Decision Tree (DT), SVM, Back Propagation neural network (BPNN) in the landslide susceptibility assessment. Huang and Xiang [36] have found that the deep learning method deep belief network has a comparable classification accuracy to BPNN when landslide points were more than 1000. In addition, in the landslide susceptibility study, the identification of landslide points is one of difficult problems, which often requires manual detection in the field and costs a lot. The high-precision image classification ability with deep learning method can help identify the research area to make the experiment more convenient. In the next phase of work, we will focus on the application of deep learning in landslide susceptibility assessment.
